# The Regulation of Host Intestinal Microbiota by Polyphenols in the Development and Prevention of Chronic Kidney Disease

**DOI:** 10.3389/fimmu.2019.02981

**Published:** 2020-01-08

**Authors:** Naren Bao, Fangjie Chen, Di Dai

**Affiliations:** ^1^Department of Anesthesiology, The First Hospital of China Medical University, Shenyang, China; ^2^Department of Medical Genetics, School of Life Sciences, China Medical University, Shenyang, China; ^3^Department of Laboratory Medicine, The First Hospital of China Medical University, Shenyang, China

**Keywords:** polyphenols, gut health, chronic kidney disease, inflammation, intestinal microbiota

## Abstract

Polyphenols are essential antioxidants in our regular diet, and have shown potential antibacterial effects. Other important biological effects, such as anticancer or antibacterial activities, have been demonstrated by some polyphenols. In recent years, the benefits of polyphenols to human health have attracted increasing attention from the scientific community. Recent studies have shown that polyphenols such as anthocyanin, catechin, chlorogenic acid, and resveratrol can inhibit pathogenic bacteria such as *Escherichia coli* and *Salmonella* to help regulate intestinal microflora. An imbalance of intestinal microflora and the destruction of intestinal barrier function have been found to have a potential relationship with the occurrence of chronic kidney disease (CKD). Specifically, they can aberrantly trigger the immune system to cause inflammation, increase the production of uremic toxins, and further worsen the condition of CKD. Therefore, the maintenance of intestinal microflora and the intestinal tract in a stable and healthy state may be able to “immunize” patients against CKD, and treat pre-existing disease. The use of common antibiotics may lead to drug resistance in pathogens, and thus beneficial polyphenols may be suitable natural substitutes for antibiotics. Herein we review the ability of different polyphenols, such as anthocyanin, catechin, chlorogenic acid, and resveratrol, to regulate intestinal microorganisms, inhibit pathogenic bacteria, and improve inflammation. In addition, we review the ability of different polyphenols to reduce kidney injury, as described in recent studies.

## Introduction

Chronic kidney disease (CKD) affects over 10% of the population and is increasingly pervasive in several nations, largely due to aging populations and changing lifestyles (1). Disease presence and occurrence vary due to differences in people's pre-existing underlying conditions and access to government-subsidized care. In many countries, the frequency of CKD is as high as 200 cases per million per year (2).

CKD is characterized by chronic renal damage and/or functional impairment, and it is often associated with high rates of morbid obesity and mortality throughout its progression, from early to advanced stages, at which point renal replacement therapy (i.e., dialysis or transplant) is required. Although, significant progress has been achieved in the prevention, detection, and treatment of CKD, it remains a significant public health problem (3), and there is therefore an immediate need to develop new approaches to treat patients with CKD (4).

The human intestinal tract is the location of a diverse bacterial community denoted the intestinal microbiota. It has a key role in preserving the well-being of humans, but it is vulnerable to external factors such as dietary patterns and the use of medications (5). The microbiota retains a codependent relationship with its human host in normal conditions, but its dysfunction has been linked with a number of diseases. For example, intestinal microbial dysbiosis occurs in CKD, manifesting as an increase in pathogenic flora relative to symbiotic flora (5). CKD has also been observed to increase the permeability of the intestinal barrier, leading to the increased passage of endotoxins and other bacterial substances into the blood (6).

In a specific example, an accumulation of uremic contaminants is usually associated with an increased risk of CKD development. Some uremic toxins or their precursors are generated by the processing of nutrients by intestinal microbiota, where these precursor are typically trimethylamine N-oxide (TMAO), indole-3-acetic acid, *p*-cresyl sulfate, and indoxyl sulfate. A higher intake of certain nutrients may also affect the intestinal microbiota, and lead to the increased bacterial production of uremic toxins or precursors. Thus, circulating concentrations of nutrient-derived uremic contaminants are associated with an increased risk of CKD development (7).

Dietary polyphenols found in a wide range of plant foods have been shown to be correlated with beneficial effects in the hosts (8). Polyphenols can also help to prevent chronic diseases such as cardiovascular disease, hypertension, obesity, and neurodegenerative diseases (1). Studies have shown that dietary polyphenols can help to maintain bowel health by promoting the development of beneficial bacteria (i.e., *Lactobacilli* and *Bifidobacteria*), thereby maintaining microbial gut equilibria, and by inhibiting pathogenic bacteria, thus demonstrating polyphenols' positive prebiotic effect (8).

In this article we review the evidence in the literature of a link between intestinal health and CKD, with a special focus on the effects of polyphenols on the regulation of intestinal microbial communities and immune responses for the prevention and treatment of CKD.

## Polyphenols

### Definition and Dietary Sources

Polyphenols are a class of phytochemicals with the strongest presence in plants. They are the products of secondary plant metabolism via two fundamental metabolic pathways: the shikimate pathway and the acetate pathway. Polyphenols are categorized as flavonoids or non-flavonoids, and are present in two main structural forms: bound to sugars to form glycosides, which have enhanced solubility, or as free, non-sugar-bound species, known as aglycones (9). Polyphenol aglycones are a heterogeneous molecular group, classified into four main classes according to their chemical structure: flavonoids (e.g., flavonols, flavanols, flavanones, anthocyanidin, chalcone, dihydroflavonoles, and isoflavone), lignans, stilbenes, and tannins [e.g., phenolic acids, such as hydroxybenzoic, hydroxygenylpropanoic, and hydroxybenzoic acids (5)].

The potential health benefits of polyphenols and their possible ability to help prevent cancer and cardiovascular diseases have made them a subject of intensive research. Polyphenols have demonstrated health-enhancing effects in anticarcinogenic, antimicrobial, vasodilatory, and analgesic processes (10). Mechanistically, the *in situ* intestinal processing of polyphenols by microbes in the human intestine yields potentially bioactive low-molecular-weight metabolites, which are the putative health-enhancing species (11).

### Bioavailability of Dietary Polyphenols

There are different ways to define bioavailability, and the commonly accepted conceptualization is that it represents the proportion of nutrients that are digested, absorbed and metabolized by normal biochemical pathways. It is therefore important to know not only how many foods or dietary supplements contain a given nutrient, but also how much of this nutrient is bioavailable (12). Various polyphenols, such as flavonoids and phenolic acids, have been found to reduce the absorption of minerals such as iron, zinc and copper species, and other trace components, most likely due to chelation, e.g., by galloyl and catechol hydroxyl groups. For example, polyphenols like epigallocatechin gallate (EGCG) and other gallates appear to negatively affect iron and zinc absorption in Caco-2 (colon cancer) cells (13). The effect of the intake of minerals on the bioavailability of polyphenols has been thoroughly evaluated, except for the more controversial effect on zinc (14).

In the past, the concept of a prebiotic applied only to carbohydrates, but recent evidence has led to a new definition: “a prebiotic is a non-ingestible compound that modulates the structure or function of gut microbiota through its metabolization by microorganisms in the intestines, thereby giving the hosts a physiologically beneficial effect.” Crucially, this new definition includes polyphenols as relevant microbiota modulators (15). A human study found that red wine polyphenol consumption increased the levels of *Enterococcus, Prevotella, Bacteroides, Bifidobacterium, Bacteroides uniformis, Eggerthella lenta*, and *Blautiacoccoides* in the rectale group in the intestine. Meanwhile, the quantity of *Lactobacillus* spp. was unchanged. Moreover, the development of pathogenic bacteria such as *Clostridium difficile, Clostridium perfringens*, and *Bacteroides* spp. was significantly constrained by the treatment of cultures with different tea phenols (16). In addition, while anaerobes such as *Bifidobacterium* and *Lactobacillus* are less affected, Vendrame et al. observed a significant increase in the quantity of *Bifidobacterium* after the consumption of beverages containing wild blueberries, indicating that wild blueberry-derived polyphenols play a key role in the regulation of intestinal microbiota composition (16).

### Anthocyanins, Catechins, Resveratrol, and Chlorogenic Acid

#### Anthocyanins

Anthocyanins are water-soluble plant pigments that exhibit intensely vibrant violet, red, or blue colors. The comprise a primary subclass of polyphenol flavonoids, and epidemiological studies have shown that they can improve a diverse range of health indicators, such as vision, blood pressure, and cognition, as well as protect against risk factors for heart disease (17).

The growth of *Bifidobacterium* spp. and *Lactobacillus-Enterococcus* spp. have been found to be widely and significantly enhanced by anthocyanins, indicating that anthocyanin metabolites support intestinal bacterial community members (11). Studies have shown that dietary anthocyanins obtained from fruits and vegetables can protect against bowel inflammation and offer other colon health benefits. Thus, anthocyanins are active in maintaining intestinal mucosity, restoring the epithelial barrier structure, immuno-modulating and controlling the microbiota, which combine to provide anti-inflammatory benefits (18, 19). In a model of DSS-induced murine colitis, a 2-week diet of cooked black bean (20% consumption) dramatically blocked colon shortening and spleen enlargement (20). Bibi et al. found that red raspberry anthocyanins exhibited protective barrier activity in the intestine; specifically, they significantly inhibited the elevation of claudin-2 protein and increased the expression of claudin-3 and ZO-1 under dextran sulfate sodium (DSS) treatment (21).

These results show that anthocyanins are able to protect the intestinal barrier by modulating tight junction-positive and -negative protein ratios, exemplifying the anti-colonic inflammation effects of anthocyanins from various fruits and vegetables (22, 23). In addition, anthocyanins have high antioxidant abilities, attributable to the ability of their phenolic groups to donate electrons, or transfer electrons from hydrogen atoms, to quench free radicals. Moreover, anthocyanins have shown anti-estrogenic, anti-inflammatory, cell proliferation-inhibiting abilities, and the capacity to reduce lipid accumulation during adipocyte differentiation, further demonstrating their ability to decrease disease risk (24).

#### Catechins

Catechins are volatile and can be easily degraded and metabolized by interactions with hydroxyl groups on phenol rings under physiological conditions. Even when administered intravenously, catechins are partly degraded before they reach the target tissues (25). Catechins also undergo metabolic breakdown in the liver, small intestine, and colon (26). Several recent studies have described the anti-infective characteristics of the primary catechin of green tea leaves, epigallocatechin-3-gallate (EGCG). Several authors have observed that EGCG blocks the entry of the hepatitis C virus by impairing viral attachment to the cell surface (27). EGCG has also been found to block cell invasion by multiple viruses and exhibit anti-HIV-1 viral effects at several points in the viral lifecycle (28). It has also been shown that catechins and tea epigallocatechins have a protective effect in gastrointestinal ailments such colitis and colon cancer (5). The antimicrobial activity of green tea catechins (GTCs) has been widely researched, and green tea has been shown to function synergistically with various antibiotics in many ways, directly and indirectly, against these pathogens. Antimicrobial activity can also be derived from the anti-inflammatory and antioxidant properties of green tea (26).

During carcinogenesis, the diversity and count of microbiota are enriched, *Bacteroides* spp. thrive and the abundance of butyrate-producing bacteria, such as *Clostridiaceae* and *Ruminococcu*s, decreases continuously (29). In comparison, EGCG treatment during colon carcinogenesis maintains a relatively stable intestinal microbiota composition (29). This tumor-suppressive effects of EGCG may be attributable to its enhancement of the accumulation of beneficial microbes such as *Bifidobacterium* (29). Thus, EGCG and other dietary components affects the intestinal microbiota. In addition, EGCG exhibits antioxidant behavior and epigenetic effects, such as inhibiting DNMT1 to block CpG methylation, thus re-activating silenced genes, such as tumor suppressors (30). These results jointly indicate the protective effects of epicatechin against high-fat diet (HFD)-related increased intestinal permeability and endotoxemia, and against cancer. This also partly explains epicatechin's ability to prevent steatosis and insulin resistance in people who consume an HFD (31). Further studies are needed to investigate the molecular mechanisms of bioactive catechins (32).

#### Resveratrol

Resveratrol is an antifungal and antibacterial stilbenoid derived from plants and can be obtained from diets consisting of various fruits such as grapes and their juice, oranges and cranberries, red currants and peanut skins (33). In both *in vitro* and *in vivo* studies, resveratrol has proved to be an effective antioxidant, antibacterial, anti-obesity, anti-inflammatory, and anti-cancer agent (34–36). Resveratrol is generally thought to be an efficient scavenger of reactive oxygen species (ROS) and free radicals. Moreover, Manach et al. suggested that the main nutrient antioxidants in the colon are polyphenols because the small intestine utilizes other antioxidants such as vitamins E and C. Khader et al. also reported novel resveratrol analogs that may alleviate ischemia-reperfusion-induced renal damage by the regulation of IL-1, IL-6, and TNF-α levels, and the modulation of the host energy metabolism (37). In contrast, Bienholz et al. reported that resveratrol itself did not ameliorate ischemia-reperfusion-induced acute kidney injury in rats (38).

Although the effect of resveratrol on host metabolism has been shown to be positive, analyses of the plasma concentrations have indicated that resveratrol has low bioavailability, as it is too rapidly metabolized to be an effective orally administered medication. Hepatic and gut microbial enzymes can metabolize resveratrol (34), and, surprisingly, numerous studies have suggested that the composition of intestinal microbiota is modulated by resveratrol, and that this may account for its anti-obesity activity and metabolic benefits. Resveratrol intake (200 mg/kg/day) effects were first demonstrated in an overweight HFD mouse model, where a marked improvement in gut microbial dysbiosis and reduced fat/body weigh were observed. Impressively, the counts of *Lactobacillus* and *Bifidobacterium* and the levels of Bacteroidetes and Firmicutes–which usually decrease in obese animals—significantly increased, while the concentration of *Enterococcus faecalis* decreased (36).

Other research has revealed that resveratrol can enhance the integrity of cell junctions by upregulating the expression of intestinal tight-junction proteins such as tight-junction protein 2 (39) and occludin. In addition, resveratrol has been shown to facilitate the assembly of close junction protein claudine 4, which may reverse the increase in intestinal permeability caused by the widespread mycotoxin deoxynivaenol. Given the role of resveratrol in maintaining an intact intestinal barrier and the “leaky gut” pathophysiology present in several mechanisms of disease, this research avenue warrants further investigation (33).

Finally, the dietary use of resveratrol is partly able to ease the adverse effects of heat stress in broilers by restoring the damaged structure of the villus-crypt in the intestinal barrier, altering intestinal microflora profiles and increasing the mRNA expression of genes related to tight- and adherence-junctions of the gut (35).

#### Chlorogenic Acid

Chlorogenic acid (3-*O*-caffeoyl-quinic acid, C-QA) is the caffeic ester of quinic acid, and one of the most abundant phenolic acids in the Western diet. It was found that some C-QA bypasses absorption in the small intestine and travels to the colon, where it is converted into several metabolites by the resident microbiota (40). C-QA administration (150 mg per kg daily) significantly increased body fat depletion, decreased lipid levels in plasma and altered levels of mRNA of lipogenesis and lipolysis related-genes in adipose tissues. In addition, C-QA reversed gut microbiota dysbiosis caused by an HFD, substantially inhibiting the growth of Desulfovibrionaceae, Ruminococcaceae, Lachnospiraceae, Erysipelotrichaceae, and boosting the growth of Bacteroidaceae and Lactobacillaceae.

The C-QA-generated amelioration of HFD-induced dysbiosis in gut microbiota may partly explain its beneficial effects on HFD-induced obesity (41). Moreover, C-QA therapy has been shown to reverse the deleterious effect of lead ion (Pb^2+^)-induced intestinal microbiota compositional changes. For example, the proportion of *Helicobacter* increased from 2.95% (Pb^2+^) to 11.24 % (Pb^2^ + + C-QA) and the proportion of *Lachnospiraceae* NK4A136 group decreased from 7.09% (Pb^2+^) to 2.68% (Pb^2+^ + C-QA) (42). This suggests that C-QA is a natural product capable of reversing Pb^2+^-induced nephrotoxicity and hepatotoxicity.

In a DSS-induced colitis mouse model, C-QA acted on the bacterial population to modify intestinal structure, leading to lower intestinal and systemic inflammation and increased healthy progression, related to a proportional increase in *Akkermansia* (43). Moreover, C-QA reduced diversity loss in the composition of fecal microbiota in ACTH-treated rats (44). C-QA enhanced microbiota diversity at the genus and order level: pre-treatment with C-QA increased the relative abundance of certain primary bacteria in the gut, such as Desulfovibrionales, Desulfovibrio, and Burkholderiales, as well as *Klebsiella* and *Bifidobacterium*.

## Intestinal Microbiota

### Gut Microbiota

The body's gastrointestinal system is colonized by an extremely large number of microorganisms, primarily bacteria. It has been calculated that the human adult microbiota comprises ~10^14^ bacterial cells, equivalent to ~10 times the total average number of human cells. Meanwhile, the metabolic capacity of the intestinal microbiome is ~10 times greater than the liver's metabolic abilities (45). The host organism and the intestinal microbiota change and develop together, executing a wide range of immunogenic, and metabolic interactions (46–48). Commensal bacteria promote cell generation, growth, and maturity within the intestine, improve immune functions and provide a defense against enteric pathogens (49). Indeed, the intestinal microbiota plays a crucial role in preventing and protecting the host organism from infection, as it inhibits the spread and colonization of pathogens. Moreover, intestinal microbiota assist in the process of native immune-cell differentiation and development (46).

Advanced metagenome research, such as DNA sequencing-based studies used for microbial ecology analysis, has identified strong correlations between intestinal microbiota and human disease. Knowledge of the intestinal microbiome and various microbial functionalities has accumulated in recent decades, primarily generated from large-scale research projects such as the Human Microbiome Project (HMP), the European Metagenomics of the Human Intestinal Tract (MetaHIT), integrative HMP (iHMP), and MetaGenoPolis. As technology and clinic-based care become more interconnected, it will become possible to monitor and assess an individual's health on the basis of his/her intestinal microbiome data (50).

In addition, intestinal microbes are involved in the physiology of the gastrointestinal tract, and changes in their relative population concentrations can damage the beneficial relationship between microbiota and host organism, and have a direct effect on health (51). Over the last decade, researchers have emphasized several key dimensions of the mammalian host–gut microbial interaction. The microbiota plays a very significant role in human metabolism, and is now regarded as a metabolizing “organ” with effects on endo- and xenobiotic catalysis, carbohydrate metabolism and on other key chemical and immunological processes (52).

### Dysbiosis of Gut Microbiota

The effect of the intestinal microbiota on human health merits more attention given that significant differences in the composition of intestinal bacteria or the gut itself are seen in subjects with inflammatory bowel disease, autoimmune diseases, allergies, and lifestyle-related illnesses (53). Notably, the dietary-influenced composition of microbiota is not consistent throughout a person's life; for example, it is normal for the diet of elderly people to change due to decreasing taste and smell perception, tooth loss and chewing problems, or because illnesses necessitate the removal of certain components from their diets (45).

In the last 50 years, many sociocultural developments have brought new, greater disturbances to the human gut microbial ecology, as the modernization of the Western industrial lifestyle has led to the increased intake of antibiotics and components from the production of meat-based and hygiene goods (e.g., emulsifiers and sweetening agents). Complex habitats such as the bacterial intestinal ecosystems can maintain only a few balanced equilibrium states (54), and when these delicate equilibria are strongly perturbed, host immune cells target resident bacteria. In people with certain genetic defects in immune response control, this can create chronic bowel inflammation (53). In another example, research comparing human host insulin sensitivity, the fasting serum metabolome, and the intestinal microbiome with findings from mice studies has indicated that specific bacteria can cause resistance to insulin (55).

Additionally, the early composition of microbiota in preterm infants has been shown to be an indicator of the future occurrence of necrotizing enterocolitis (56). Animal studies have also demonstrated that the early microbiota composition is a key driver of the correct subsequent development of homeostasis, and of the host as a whole (5). Inflammatory bowel disorders (IBDs), inflammatory skin diseases such as psoriasis and atopic dermatitis, auto-immune arthritis, type 2 diabetes, obesity, and atherosclerosis have been found to be associated aberrant structure and function of the intestinal microbiota (57).

### Polyphenols Alter the Gut Microbiota Composition

Some phenolic compounds with bacteriostatic and bactericidal activity have been considered as potential antimicrobial agents. It has been shown that these can be used to prevent bacterial infections in the stomach and urinary epithelium (11). The effects of common polyphenols on the growth of human intestinal bacteria and their adherence to enterocytes were investigated by Parkar et al. (58). The *in vitro* viability of representative gut microflora was found to be affected by doses of polyphenols equivalent to concentrations likely to be present in the gastrointestinal tract, but these effects depended on the type of polyphenols analyzed, and did not include rutin (45).

Polyphenol-rich cocoa decreased the Firmicutes/ Bacteriodetes ratio and increased *Faecalibacterium prausnitzii* levels in pigs (59). The countering of intestinal microbiota dysbiosis in rats fed with an HFD and sucrose diet inhibited body weight gain, decreased serum insulin, reduced the Firmicutes/Bacteriodetes ratio, and restricted bacterium production associated with lifestyle-associated obesity (60, 61). A relative increase in the abundance of bactericides in subjects drinking red wine polyphenols has also been reported (62).

*Bifidobacterium* is a widely used probiotic strain with documented health benefits in areas such as immuno-modulation, cancer prevention, and IBD control (57). Vendrame et al. found a significant rise in *Bifidobacteria* in subjects who drank a wild blueberry-based beverage, showing the important role of polyphenols within wild blueberries in the regulation of the intestinal microbiota structure and environment (16). Table 1 provides a summary of recent studies on the effects of various polyphenols on intestinal health.

**Table 1:** Recent studies on the effects of different polyphenols on intestinal health.

**Type of polyphenols**	**Source of polyphenols**	**Effects**	**Potential applications**	**References**
Anthocyanins	Turkish *Cinclidotus fontinaloides*	Imparted resistance to *Salmonella*	In medicine, cosmetics, and pharmaceutical applications as well as in food and agriculture	(63)
Anthocyanins	Seed coats of selected soybean varieties	Imparted resistance to *Escherichia coli*	For reducing levels of foodborne bacteria contaminating poultry products	(64)
Anthocyanins	Black rice	Prebiotic-like activity by the modulation of the intestinal microbiota	Modulation of the intestinal microbiota	(65)
Anthocyanins	Purple sweet potato	Induced the proliferation of *Bifidobacterium bifidum*, prebiotic-like activity through the modulation of the intestinal microbiota	Modulation of the intestinal microbiota	(66)
Anthocyanins	Black raspberry	Lowered the levels of DNMT31 and DNMT3B, as well as of p-STAT3	Modulation of the composition of gut commensal microbiota	(67)
Anthocyanins	Blackcurrant	Increased the number of goblet cells in the colon	Alteration of the biomarkers of large intestinal health	(68)
Anthocyanins	Blueberries	Improves markers of insulin sensitivity	Generation of compositional changes in the gut microbiota associated with improvements in systemic inflammation and insulin signaling	(69)
Anthocyanins	Black raspberries	Increased levels of butyrate-producing bacteria, e.g., Anaerostipes		(70)
Catechins	Green tea	Inhibited intestinal OATP1A2-mediated uptake of sulphobromophthalein	Reduction of plasma concentrations of nadolol	(71)
Catechins		Altered gut microbiota and gene expression and function in colonic epithelial cells	Induction of host weight loss	(72)
Catechins	Green tea	Decreased the number of precancerous lesions as well as solid tumors	Inhibition of colon carcinogenesis	(29)
Catechins	Green tea	Reduced species richness and abundance of gut bacteria in *E. obliqua* larvae	Promotion of larval fitness associated with ECGG antimicrobial activity	(73)
Catechins	Parapiptadenia rigida	Imparted resistance to *Escherichia coli*	Treatment of infectious diarrhea	(74)
Catechins	Common herbs	Imparted resistance to *Escherichia coli*	Treatment of infectious diarrhea	(75)
Curcumin and resveratrol		Regulated gut microbiota of weaned piglets, downregulated the TLR4-signaling pathway	Alleviation of intestinal inflammation, and ultimately increased intestinal immune function	(37, 76)
Resveratrol		Restored the impaired villus-crypt structure, modified the profiles of intestinal microfloras, and altered the mRNA expression of intestinal tight junctions- and adherence junctions-related genes in broilers	Amelioration of the adverse effects of heat stress on intestinal barrier function in broilers	(35)
Chlorogenic acid		Increased the diversity of gut microbiota	Regulation of the gut microbiota and increase of serum free amino acid levels	(77)
Chlorogenic acid		Ameliorated HFD-induced gut microbiota dysbiosis	Amelioration of HFD-induced obesity	(41)
Chlorogenic acid		Modified the gut microbial community structure	Antidepressive effects	(44)

## Chronic Kidney Disease

### Causes

CKD is characterized by an unusual renal structure or activity with health consequences spanning more than 3 months. Indicators include albuminuria, irregular urinary debris, abnormal vision, serum electrolyte and pH changes and a glomerular filtration rate (GFR) <60 ml.min^−1^ relative to a normal body surface area of 1.73 m^2^ (78).

CKD is a major risk factor for cardiovascular disease (CVD). Uremic conditions such as CKD are treated as a consequence of aggregation of so-called uremic retention solutes (URSs), usually released from the kidneys as organic waste materials. URSs derived from phenols and indoles exist along the gastrointestinal tract (GIT), and intestinal microbiota have been shown to have be crucial in preventing detrimental effects of USRs on the cardiovascular system (79).

Hypertension, leading to renal arteriolar sclerosis and nephrosclerosis, is another important risk factor for CKD. Increasing evidence has shown that the intestinal microbiota can affect arterial blood pressure. In addition, experiments involving the transplantation of fecal material from hypertensive rats to standard-stretched mice and rats have revealed a causal relationship between gut dysbiosis and hypertension in normotensive animals. Hypertension may therefore be another gut dysbiosis mediated cause of CKD (80).

Chronic kidney failure is generally associated with aging, diabetes, asthma, obesity, and cardiovascular diseases in the developed world, with diabetic glomerulosclerosis and hypertensive nephrosclerosis being the suspected pathological entities. However, precise diagnosis is often difficult (2). Renal hypoplasia/dysplasia, posterior urethral valve problems, and other congenital kidney-or urinary tract abnormalities are the most severe etiologies. Other etiologies include polycystic kidney disease, cortical necrosis, and renal vascular thrombosis (81). CKD is usually detected in many patients during diagnostic screening procedures for other diseases. Declines in kidney function cause central and peripheral neural activity changes, due to synergistic pathway defects such as vascular calcification and urinary toxin increases both in humans and in rodents, leading to many neurological complications (82).

### Immunity

#### Gut Microbiota and Inflammation

Current evidence suggests that the host–microbiome relationship is bidirectional in people and animals with CKDs. Changes in microbiosis and function lead to excessive levels of uremic contaminants (e.g., indoxyl sulfate, *p*-cresyl sulfate, and TMAO) and decreases in levels of protective metabolites, and these changes are implicated in oxidative stress, uremia, inflammation, and deterioration of kidney function, and increased incidence of cardiovascular risk and mortality (83).

A variety of proteinuric kidney diseases such as IgA nephropathy and lupus nephritis are associated with microbial dysbiosis. By regulating the activity of regulatory T cells (Treg), intestinal microbiota can influence the development of idiopathic nephrotic syndrome, which has been shown to be alleviated by increased Treg activity. In addition, the development of renal amyloidosis may be facilitated by the chronic inflammation caused by intestinal dysbiosis, as this contributes to the production of chronically regulated serum amyloids A (SAA) (80). Levels of claudin-1, occluding, and ZO-1 protein are excessively reduced in the colonic mucosa of animals with CKD, as shown by Vaziri et al. (84). This suggests that the disruption of the colonic epithelial tight-junction could lead to bacterial and endotoxin translocation across the intestinal wall, generating the systemic inflammation typical of CKD. A damaged bowel barrier structure has been shown to enable the transmission of uremic toxins, contributing to an accumulation of these toxins, due to the compromised excretory process (85).

In addition, as proteobacteria may cause inflammation by damaging intestinal barrier functions and generating pro-inflammatory compounds such as uremic metabolites and serotonin, an increased Proteobacteria count may contribute to the pathogenesis of a number of chronic inflammatory diseases, and thus lead to proteinuric nephropathy (80).

#### Gut Microbiota and Immunity

When analyzing the causes of CKD and the factors affecting its progression—such as type 2 mellitus (T2DM), overweightness and hypertension—it can be concluded that lifestyle changes can directly and positively affect the natural course of CKD (1). Post-natal bacterial bowel colonization stimulates the immune system and decreases sensitivity to food and toxins. Human immune regulation of the microbiota is best demonstrated by a gut microbial human-immune maturation induction experiment. In this manner, different species of microbiota have been shown to cause levels of forkhead protein 3-expressing Tregs to increase, or to differentiate. Some of these Tregs are active in microbial antigen detection, and in germfree mice with a number of specified benign commensals, levels of colonic Tregs also increase (85). Cumulative analyses and experiments have shown that some microbial species of human intestinal microbiota are correlated with multiple physiological disorders, including those with effects on human health. The *Bifidobacteria* in breast-fed children are a typical example of this association.

Thus, the protective effect of nutritious feeding and numerous studies on immune disorders and diseases have been demonstrated (51). Host genes and immune function also influence gut microbiota composition. To date, the human genes believed to affect the structure of the intestinal microbiota have been mostly those coding for proteins of the immune system (86).

### Gut Microbiome in Kidney Disease

Intestinal dysbiosis, typical in CKD subjects, results from qualitative and quantitative modifications of the host microbiome profile and resultant effects on the intestinal barrier. Increased CKD, increased cardiovascular risk, uremic toxicity, and inflammation are associated with modifications to the microbiota and the subsequent diverse host responses (85). Experimental and clinical data from genomic and non-genomic studies have suggested that an unusual composition of microbiota exists in CKD (7), and preliminary results have suggested that the composition of the microbiota in chronic renal failure patients and early stages of CKD may be altered (87). More recently, the accumulation of uremic toxins was shown to be due to a number of microbiota strains. For example, the breakdown of tyrosine and phenylalanine by variety of obligate or non-obligate intestinal anaerobics such as the genera Bacteroides, Lactobacillus, Enterobacter, Bifidobacterium, and in particular Clostridio leads to the generation of *p*-cresyl from its conjugate *p*-cresyl sulfate (PCS) (88).

In 1965, Einheber and Carter found that anephric mice with an unaltered intestinal microbiome survived longer than anephric mice (85). There is now extensive evidence that the intestinal barrier is damaged in CKD, leading to the translocation of uremic toxins originating from bacteria and other noxious luminal products into the bloodstream, resulting in inflammation, and the activation of leukocytes. Gas chromatography studies in CKD rats and end-stage kidney disease (ESKD) patients showed significant changes in exhaled breath gases compared with those of healthy controls, further evidencing the altered gut microbe composition in these patients (89).

The gut microbiome may also secrete useful kidney-preserving metabolites such as short-chain fatty acids (SCFAs) (86), which can reduce CKD injuries. For example, in research by Wang et al., butyrate levels were approximately three times higher in the healthy controls than in CKD patients. They also showed that mice transplanted with fecal microbiota obtained from CKD subjects showed accelerated CKD progression, in the form of higher levels of TMAO, and that this could be reversed by additional butyrate supplementation (90). Thus, delayed CKD progression may be mediated SCFA produced by intestinal microbiota.

### Polyphenols, Microbiota and CKD

The accumulation of uremic toxins is related to the increased risk of CKD development. Many uremic toxins are derived from the absorption of nutrients by gut microbiota, as these nutrients enhance bacterial growth, which in turn produce more uremic toxins. Thus, possible treatments can involve diet modification, changes in microbiota, reducing the production of uremic toxins by microbiota, increasing the excretion of toxins or targeting the removal of certain uremic toxins (7). Of these, diet is the most powerful and easily controlled factor in intestinal microbiota modulation. To date, however, no research on the impact on the gut microbiota profile of CKD has been undertaken, and most related questions remain unanswered.

The bioactive compounds in foods like polyphenols, and their potential to modulate intestinal microbiota, have been given more recent attention. These compounds, which are powerful antioxidants and natural anti-inflammatory agents, are widely used to prevent inflammation and oxidative stress in chronic “burden of lifestyle” illnesses (91). Several studies have confirmed the capacity of probiotics to control gastrointestinal disease, and the success of treating CKD patients with *Clostridium difficile* infection should be paid particular attention in this regard (79). The findings of various studies, which have been based on a number of laboratory models, including *in vitro* experiments, *in vivo* experiments, and clinical studies, have reinforced the support for the health-promoting properties of polyphenols. Table 2 summarizes various types of polyphenol effects on CKD found in recent years.

**Table 2:** Effects of various types of polyphenols on CKD observed in recent years.

**Type of polyphenol(s)**	**Mechanism of action**	**Effects**	**References**
Resveratrol	Suppressed the NF-κB signaling pathway	Attenuated skeletal muscle atrophy in CKD mice	(92)
Resveratrol	Inhibited the NLRP3 inflammasome and IL-1β secretion	Protected the kidney against tubulointerstitial injury	(93)
Resveratrol	Suppressed the Ang II/AT1R axis and enhanced the AT2R/Ang 1-7/MasR axis function	Exerted protective effects on aging kidneys	(94)
Resveratrol	Reduced the lipopolysaccharide-induced inflammatory response	Ameliorated sepsis-induced acute kidney injury in a pediatric rat model	(95)
Resveratrol	Inhibited the endoplasmic reticulum stress-activated NF-κB pathway	Protected against early polymicrobial sepsis-induced acute kidney injury	(96)
Procyanidine	Enhanced GFR and markedly decreased proteinuria	Improved kidney function	(97)
Anthocyanins	Reversed diabetes-induced increases in renal apoptosis and oxidative stress	Ameliorated diabetic nephropathy function parameters in db/db mice	(98)
Anthocyanins	Abated the effects of adenine-induced CKD	Antagonized oxidative stress and inflammatory reactions associated with CKD	(99)
Anthocyanins	Disturbed the Angpt-Tie-2 ligand-receptor system linked to renal VEGFR2-signaling pathway	Antagonized glomerular angiogenesis due to chronic hyperglycemia and diabetes	(100)
Catechins	Exhibited antioxidant, anti-inflammation, and mitochondrial protection	Effectively protected the kidney against the toxic effect of cadmium	(101)
Catechins	Exhibited antioxidant and possible direct nephroprotective actions	Prevent gentamicin-induced experimental nephrotoxicity	(102)
Catechins	Scavenged ROS and maintained near-control levels of antioxidant enzyme activities	Prevented vanadium-induced lipid peroxidation and nephrotoxicity in an experimental model	(103)
Catechins	Inhibited TLR4 upregulation and NOX activation and the consequent downstream events, e.g., NF-κB activation	Prevented lipopolysaccharide (LPS)-induced renal inflammation	(104)
Catechins	Prevented NF-κB activation and upregulated NADPH oxidase 4 (NOX4) in the kidney cortex	Diminished inflammatory responses	(105)
Catechins	Disabled KEAP1 and upregulated NRF2	Prevented diabetic nephropathy	(106)
Chlorogenic Acid	Inhibited the TLR4/NF-κB signaling pathway	Attenuated LPS-induced acute kidney Injury	(107)
rutin	Reduced renal interstitial injury and suppressed interstitial collagen deposits in UUO rats	Ameliorated kidney interstitial fibrosis in rats with obstructive nephropathy	(108)
Chlorogenic acid	Regulated the dysbiosis of the gut microbiota in mice	Relieved Pb^2+^-induced cognitive impairments and hepato-renal damage	(42)

Resveratrol treatments of the CRL-2573 rat kidney cell line and mesangial primary cells prevented the fibronectin expression and the proliferation of high glucose-induced mesangial cells, respectively (109). Resveratrol decreased atrophy in skeletal muscles in chronic muscle disease via the muscle-specific ring-finger protein 1 (MURF1) signaling pathway (92). Also, it significantly reduced creatinine-mediated interstitial damage in murine CKD models (93), with resveratrol-treated mice showing better renal function and increased albuminuria, and good histological test results.

In another example, the angiotensin II (Ang II)-angiotensin II type 1 receptor (AT1R) axis was suppressed and the function of the angiotensin II type 2 receptor (AT2R)–Ang 1-7-Mas receptor (MasR) axis was enhanced by resveratrol. Additionally, the expression of nicotinamide adenine dinucleotide phosphate oxidase 4, 8-hydroxy-2′-deoxyguanosine, 3-nitrotyrosine, collagen IV, and fibronectin were decreased, while the expression of endothelial nitric oxide synthase and superoxide dismutase 2 were increased following resveratrol treatment. Resveratrol can thus have protective effects on aging kidneys, via Ang II inhibition, and MasR activation leading to decreased oxidative stress, inflammation, and fibrosis (94).

Research also showed that resveratrol decreased *in vitro* LPS-induced inflammatory effects in kidney cells, and lead to the activation of nuclear factor E2-related factor 2 (Nrf2) signaling, resulting in nuclear Nrf2 accumulation, and increased expression of Nrf2 target genes heme oxygenase (HO)-1 and NAD(P)H dehydrogenase (quinone) 1 (NQO1). This was corroborated by the induction of the expression of HO-1 and NQO1 being observed to occur subsequent to *in vitro* and *in vivo* resveratrol treatment. It is worth noting that the knockdown of Nrf2 effectively abrogated the downregulation of TNF-α, IL-1β, and kidney injury molecule-1 (KIM-1) expression resulting from *in vitro* resveratrol treatment. These findings also indicated that resveratrol improved acute kidney damage from sepsis in a pediatric acute kidney injury (AKI) model via the Nrf2 signaling pathway (95). *In vitro* studies have also demonstrated that resveratrol increased cell viability, reduced NF-κB phosphorylation, and the production of inflammatory conditions in LPS and tunicamycin-induced HK-2 cells via the restriction of inositol-requiring enzyme 1 (IRE1) activation. Resveratrol administration also immediately protected against septic AKI by inhibiting IRE1-NF-κB pathway-triggered inflammatory reactions in the kidney. Resveratrol could be thus be a rapid and elegant treatment for septic disease (96).

In cultured human glomerular endothelial cells, anthocyanins prevented high glucose-induced oxidative stress, and apoptosis through activation of AMPK. These findings suggest that anthocyanins reduce diabetic nephropathy via the phosphorylation of AMPK, the major energy-sensing enzyme, with subsequent effects on its target molecules, which seem to inhibit lipotoxicity-related apoptosis and oxidative stress in the kidney (98).

When either *H. sabdariffa* aqueous extract or the anthocyanins isolated from it were used with adenine, the detrimental impact of adenine-induced CKD was remarkably reduced, and this was mostly in a dose-dependent manner. The beneficial effects were akin to those obtained by treatment with the ACE inhibitor lisinopril (99). PCE (anthocyanin-rich purple corn extract) reduced the mesangial and endothelial induction of angiopoietin (Angpt) proteins under hyperglycemic conditions (100), and the induction and activation of VEGF receptor 2 (VEGFR2) was inhibited by treating db/db mice with PCE. These results showed that PCE antagonized glomerular angiogenesis by disturbing the Angpt-Tie-2 ligand-receptor system connected to the renal VEGFR2 signaling pathway, due to chronic hyperglycemia and diabetes. PCE could therefore be a strong, abnormal angiogenesis therapeutic agent for diabetic nephropathy that causes renal disease (100).

Reductions in levels of antioxidant thiols, superoxide dismutase, and catalase have also been found in the renal tissues of cadmium-intoxicated rats. These alterations have often been associated with mitochondrial dysfunction, supported by an increase in the production of mitochondrial ROS and a decrease in mitochondrial membrane potential. These results indicated that, probably by means of its antioxidant, anti-inflammatory, and mitochondrial defense abilities, catechin effectively protected the kidney against cadmium toxicity (101). In another example, histopathology showed degenerative changes in glomeruli and tubules 2 weeks after administration of gentamicin to rats. These renal structural and functional defects have been correlated with renal oxidative stress in such rat models, as demonstrated by the substantial, measured reductions in renal glutathione (GSH).

Catechin hydrate therapy showed significant nephroprotective action in the prevention of renal structural and functional abnormalities, and oxidative stress, in rats with gentamicin-induced nephrotoxicity (102). Oxidation was much less pronounced in ammonium metavanadate (AMV)-treated animals who received EGCG, and antioxidant enzyme activity kept close to control values. There have been other, less noticeable histopathological shifts, and taken together with those detailed above, the findings confirmed that green tea and other sources of flavonoids could provide significant defense against oxidative stress caused by ammonium metavanadate (103). Pre-treatment with epicatechin ultimately inhibited upregulation and NOX activation of toll-like receptor 4 (TLR4) and subsequent downstream events, preventing the adverse effects of LPS, e.g., activation of the NF-κB pathway.

C-QA protects liver and kidney injury in mice caused by d-galactose, due to C-QA's antioxidation and anti-inflammation effects (110). C-QA also attenuates AKI caused by LPS by inhibiting the TLR4/NF-κB signaling pathway (107). The results of this study indicated that C-QA decreased LPS-induced kidney damage by suppressing oxidative stress, inflammation, apoptosis and autophagy, enhancing kidney regeneration (111). M2/M1 macrophage polarization modulation (112) increased the ability of quercetin to reduce kidney damage and fibrosis, and treatment with quercetin improved kidney function, reduced oxidation stress factors, serum fibroblast growth factor 23 (FGF23) levels, and renal inflammation in a rat model of adenine-induced chronic kidney disease (113). Quercetin also reduced kidney damage from doxorubicin (114). Histological findings showed that rutin administration significantly reduced the interstitial lesions of renal cells and removed the deposits of UUO collagen. However, in rats with obstructive nephropathy, rutin increased interstitial kidney fibrosis (108).

Antioxidant functions in lupus nephritis (LN) was evaluated in the context of CKD. In this study, a routine dose of EGCG enhanced renal function and decreased proteinuria in a mouse model of LN (115). Furthermore, serum, urine, and kidney oxidative stress parameters in the EGCG-treated mice were considerably reduced. In a mouse model of crescentic glomerulonephritis, the therapeutic potential of EGCG was also demonstrated. This research has shown that EGCG treatment can improve renal function can be and histopathology in kidney function-impaired mice (116). The treatment also regenerated Nrf2 and its downstream products, including a catalytic subset of glutamate-cysteine ligase, a subunit modification of glutamate-cysteine ligase, and glutathione peroxidase (GPx) (116). Funamoto et al. also reported that EGCG may have a renoprotective effect after cardiopulmonary bypass in diabetic rats (117). In related work, green tea supplementation prevented the growth of rat kidney-side crystals, decreased oxalate excretion, and had inhibitory effects on the activities of *N*-acetyl-β-D glucosaminidase (118).

The prevention of the adverse effects of high fructosis (10% w/v of fructose in drinking water) in male Sprague Dawley rats in rat chow diets in 8 weeks was also observed. Dietary supplementation with epicatechin ameliorated the following ill effects in fructose-fed rats: proteinuria, decreased nephrine, synaptopodin, and tumor transcription factor (TF1) of Wilmes levels in the renal cortex, and all podocyte dysfunction indicators associated with increased oxidative stress markers, changes in activity, and expression of non-synthase (NOS) and an increased inflammatory effect (119).

## Summary and Perspectives

The intestinal microbiota has emerged as an important factor in the learning and control of the immune system, and has previously unknown functions in affecting the manifestations of many non-communicable diseases. Therefore, raising similar questions about the effects of the intestinal microbiota on CKD (120) seems logical and reasonable. In comparison, the effects of food and nutraceuticals on the uremic phenotype and its contribution to intestinal dysbiosis, given the enormous capacity of food to be used as medicine for CKD, warrant much greater attention (91).

As there is mounting evidence of the therapeutic effects of dietary polyphenols, an understanding of the *in vivo* absorption and metabolism of these compounds is increasingly important (12). In future years, meticulous analysis of these topics will be needed to exploit the potential of these research avenues.

## Author Contributions

NB finished the first draft of the manuscript. FC and DD critically revised the manuscript. All the authors approved the submission of the manuscript.

### Conflict of Interest

The authors declare that the research was conducted in the absence of any commercial or financial relationships that could be construed as a potential conflict of interest.
